# Reconstruction of Microscopic Thermal Fields from Oversampled Infrared Images in Laser-Based Powder Bed Fusion

**DOI:** 10.3390/s21144859

**Published:** 2021-07-16

**Authors:** Leigh Stanger, Thomas Rockett, Alistair Lyle, Matthew Davies, Magnus Anderson, Iain Todd, Hector Basoalto, Jon R. Willmott

**Affiliations:** 1 Department of Electronic & Electrical Engineering, University of Sheffield, Pitt Street, Sheffield S14ET, UK; leigh.stanger@nema.ltd.uk (L.S.); t.b.rockett@sheffield.ac.uk (T.R.); matt.davies@sheffield.ac.uk (M.D.); 2 Department of Materials Science & Engineering, University of Sheffield, Mappin Street, Sheffield S13JD, UK; alyle1@sheffield.ac.uk (A.L.); m.j.anderson@sheffield.ac.uk (M.A.); iain.todd@sheffield.ac.uk (I.T.); h.basoalto@sheffield.ac.uk (H.B.)

**Keywords:** quantitative thermography, thermal imaging, powder bed fusion (PBF), selective laser melting (SLM), in situ monitoring, additive manufacturing

## Abstract

This article elucidates the need to consider the inherent spatial transfer function (blur), of any thermographic instrument used to measure thermal fields. Infrared thermographic data were acquired from a modified, commercial, laser-based powder bed fusion printer. A validated methodology was used to correct for spatial transfer function errors in the measured thermal fields. The methodology was found to make a difference of 40% to the measured signal levels and a 174 °C difference to the calculated effective temperature. The spatial gradients in the processed thermal fields were found to increase significantly. These corrections make a significant difference to the accuracy of validation data for process and microstructure modeling. We demonstrate the need for consideration of image blur when quantifying the thermal fields in laser-based powder bed fusion in this work.

## 1. Introduction

Thermal imaging, or thermography, is an important and widely used tool in many Additive Manufacturing (AM) processes [1]. Ideally, spatiotemporally resolved thermal field measurements of the melt pool and Heat Affected Zone (HAZ) would be accessible. Full quantization of the surface temperature would improve our understanding of the complex phenomena occurring during deposition. A more accurate measurement of temperature would assist in the understanding of vaporization and liquation during the process and their role upon the formation of defects. The cooling rate dictates the solidification structure, and thus impacts the mechanical properties, the extent and nature of the anisotropy in mechanical properties, residual stresses, and component distortion. High-fidelity measurements are needed to verify and validate process modeling because the calculated thermal fields strongly influence microstructure and property predictions [2].

Information regarding the thermal fields is most readily accessible from the surface of the print in Powder Bed Fusion (PBF) AM processes. This is in the form of emitted thermal radiation. The quantity of emitted radiation (radiance) in one or more wavelength bands can be used to infer the temperature of the surface [3]. There are many examples that make use of measurements of radiance to infer surface thermal fields of the HAZ [4,5,6,7,8,9,10].

Accurate determination of thermal fields in the small-length-scale and high-speed process of laser-based PBF (L-PBF) is a challenging task. The main factors obscuring accurate measurement of absolute surface temperature are the emissive properties of the surface, radiation reflected from the surface, and the ability of the instrument to produce a spatiotemporally resolved map of the radiance of the surface.

The spatial resolution of a thermographic instrument is often assumed to be sufficiently high so that the images produced form a direct map of the radiance of the surface [4,5,6,7]. The size of objects that can be resolved is a combination of the optical resolution of the imaging system and the spatial sampling rate (pixel size) of the sensor. It has been shown that the combined resolution performance of the instrument can be the most significant source of uncertainty in the measured temperature for thermography on the sub-mm scale [11]. It is important to understand the performance and limitations of the instrument in its ability to reconstruct a faithful spatial map of the scene [12,13,14], and how this fidelity will affect measured thermal fields.

The standardized term for the projection of a pixel onto the scene is the Instantaneous Field of View (IFOV) [15]. This is sometimes erroneously referred to as the resolution of the thermographic instrument. The geometric projection of one pixel onto the scene can be termed a scenel. In any real-world thermographic instrument, light from surrounding scenels can be detected by the central pixel. Light originating from the central scenel can be scattered to surrounding pixels [16]. Blurring of the scene is a consequence of the inherent diffraction limit of the optics, aberrations, scatter, diffusion of charges in the sensor, and other factors, which combine to make the spatial transfer function of the instrument.

A typical measure of the imaging resolution of any imaging instrument is the spatial frequency of a sine wave in the scene, which is measured as 10% of its true amplitude by the detector (MTF = 0.1) [17]. A 90% reduction in signal is unacceptable for the purposes of accurate radiometric temperature measurements. Infrared Radiation Thermometers (IRTs) consisting of a single pixel have a more stringent measure of measurement resolution. The Measurement Field of View (Measurement FOV) of an IRT is defined as the area from inside which 95% or 99% of the radiation incident on the detector originates [18,19]. If we apply this same level of stringency to the pixels of a thermographic instrument, the Measurement FOV will be found to be substantially larger than the IFOV. Only the pixels viewing scenes that are uniform over their Measurement FOV will make an accurate measure of temperature. There does not appear to be consensus in the literature on the definition of the Measurement FOV for a thermographic instrument. We chose to use the IRT definition in this work. A consequence of this is that the effective scenels are larger than the IFOV, making direct visualization of the scene as an image problematic for accurate thermographic images.

The degree to which the signal reported by each pixel is affected by the surrounding scene is quantized by characterization of the spatial transfer function. The most significant consequence of the spatial transfer function in L-PBF is to underestimate the peak temperature, overestimate the HAZ temperatures adjacent to the laser, and to overestimate the spatial dimensions of the HAZ. The hot center of the laser heat source will significantly inflate the surrounding pixel temperature measurements up to the diameter of the Measurement FOV, obfuscating measurements of the melt pool. This problem also exists in two-color thermography [4]. Overestimation of temperature in pixels surrounding the hottest zone will be more significant in two-color than in single-band thermography due to the reduced sensitivity of the technique [20].

It is not always possible, or practicable, to design a thermographic instrument with a Measurement FOV small enough to resolve the spatial temperature gradients expected in L-PBF (0.1–20 °C µm^−1^ [5,9]). The resolution is limited by aberrations in the lens system and ultimately by the diffraction limit of the optics in optically limited (oversampled) systems. Instruments that cannot resolve the feature sizes present in L-PBF require either a method of recreating the spatial distribution of temperature or expansion of the uncertainty to take it into account. The quantification of uncertainty is itself a significant undertaking [11].

Computer processing of thermal images yields useful insight into many aspects of manufacturing; from wear on traditional power tools, to the material properties of repaired aerospace components [1,21,22]. In this work, we use computer processing, not only to directly analyze thermal images but also to provide a method for reducing the effects of the blur inherent in optical systems to give more accurate temperature measurements from these images.

For this work, we have designed a Near-Infrared Radiation (NIR) thermographic instrument and used it to capture images of the L-PBF process from a modified commercial L-PBF machine. In our attempts to quantify the thermal fields during the fabrication of the component, we encountered the classic problems of radiation thermography. We are concerned with the errors in the measurement of the thermal fields introduced by spatial resolution of the instrument. The sampling rate of the instrument is sufficient to resolve the HAZ, but the spatial transfer function of the instrument limits the measurement resolution below the Nyquist limit. The system is therefore oversampled. The Measurement FOV of the thermographic instrument is significantly larger than the HAZ of the print meaning that for accurate thermography the images must be corrected to account for this under filling of the Measurement FOV. We present in this work a method for making this correction and a method for validating corrections of this type.

Section 2 describes the details of the image acquisition, calibration, and image processing to convert from radiance to temperature. It goes on to detail the methodology applied to correct for the aberrations that limit the Measurement FOV, followed by an empirical validation test using a blackbody source of know radiance temperature.

Section 3 presents the results of the applied methodology and includes a detailed explanation of the radiance temperature calibration and further elucidates the methodology’s process and output.

**Figure 1:**
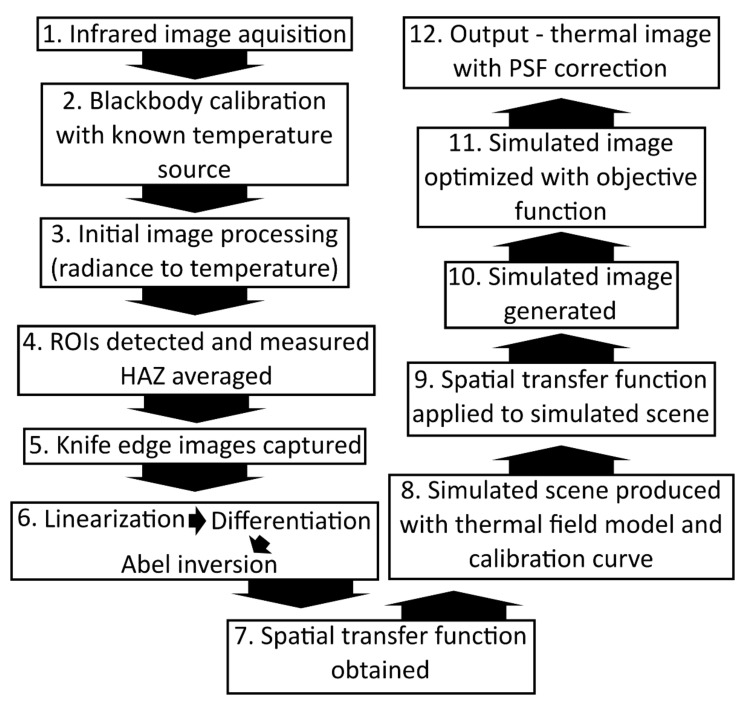
A block diagram detailing the steps involved from image acquisition to the final corrected output.

Section 4 is a discussion of the outputs from the data corrected for optical aberrations supplemented by a discussion of the results of the characterization of the optical performance of the instrument. Figure 1 shows a block diagram that shows the specific order of the steps taken in the methodology,

Finally, Section 5 contains a summation of the work and conclusions of the output of the methodology. It describes where this work could be expanded and expounds on other applications of this technique.

## 2. Materials and Methods

### 2.1. Infrared Image Collection

**Figure 2:**
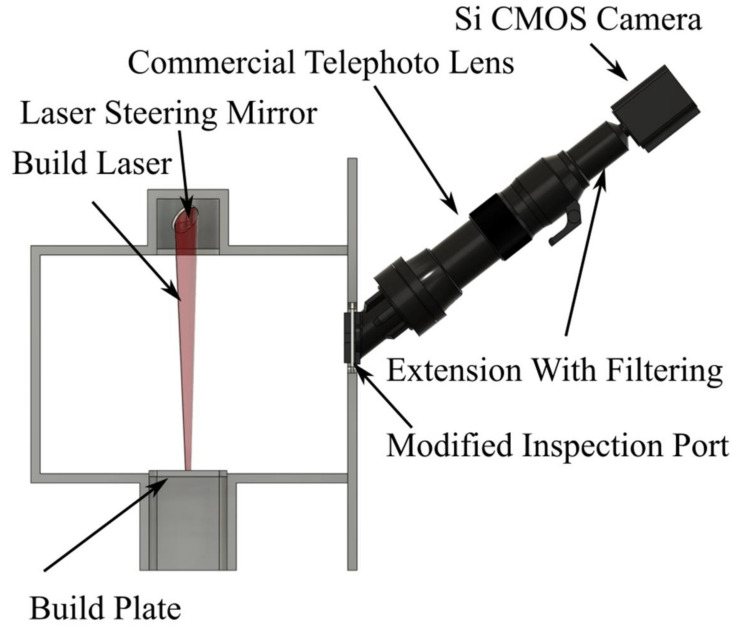
Schematic of modifications made to the Renishaw SLM 125 laser-based powder bed fusion machine to allow acquisition of infrared radiation emitted from the surface during the print process. Examples of calibrated output images from this measurement system can be seen in Figure 4.

**Figure 3:**
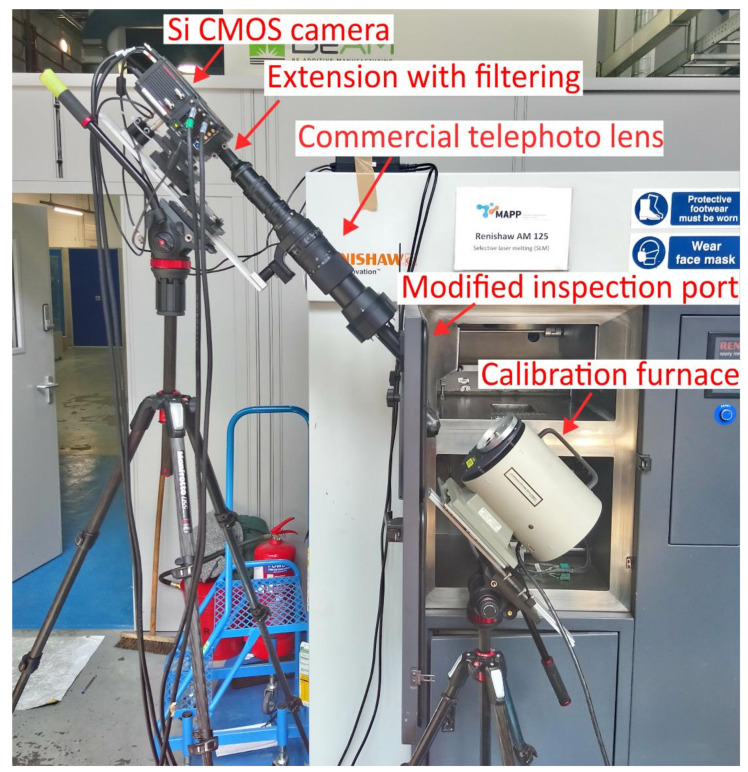
A photograph showing the setup in situ during a calibration. This system comprised two cameras whereas the system utilized in the work reported here used only one camera. The door of the Renishaw SLM 125 laser-based powder bed fusion machine is open at 90° to the machine body.

The viewport of a commercial Renishaw SLM 125 metal L-PBF machine was modified to allow unrestricted off-axis optical access, as per Figure 2. A commercial 300 mm focal length telephoto lens was used to focus an image of the build plane onto a silicon (Si) Focal Plane Array (FPA) sensor. A 16 bit, thermoelectrically cooled, Si CMOS FPA camera was used. Figure 3 shows a photograph of the setup in situ. The camera was used to acquire a series of digitally measured images of the radiance (reported in digital levels (dls) of the camera’s image output) of the surface throughout the build process. The pertinent build parameters and the imaging system properties are shown in Table 1. The maturity level of Si technology offers higher-specification instruments at a given price point than other, longer-wavelength, FPA technologies.

The sensitive wavelength range of the thermographic instrument was bounded by a 0.85 µm long-pass and a 1 µm short-pass filter. A 1.06 µm central wavelength notch filter was used to ensure that no signal was collected from the build laser of the SLM 125 machine. The short-wavelength band used in this work maximizes sensitivity to temperature while minimizing the effect of uncertainty in emissivity. The total field of view of the camera was vertically restricted to 128 pixels to allow rapid data acquisition because the image acquisition speed was limited by the line readout of the rolling shutter and so fewer rows of pixels per image increased the frame rate of the camera. The reduced image height also minimized any depth-of-field changes in the spatial transfer function due to the angle of acquisition.

The infrared measured images presented in this work were acquired throughout a single build of a 312-layer, 10 mm sided cube of the nickel-based super-alloy CM247. The build used a raster pattern with no rotation between layers.

### 2.2. Preliminary Image Processing

The measured images were acquired using the HCImage 4.3.1.30 (Hamamatsu Corporation, Bridgewater, NJ, USA) software package. All subsequent image processing and camera characterization was implemented in MATLAB R2018b (MathWorks, Natick, MA, USA). A numeric Abel inversion algorithm created for plasma physics applications was utilized in the characterization of the thermographic instrument [23].

**Figure 4:**
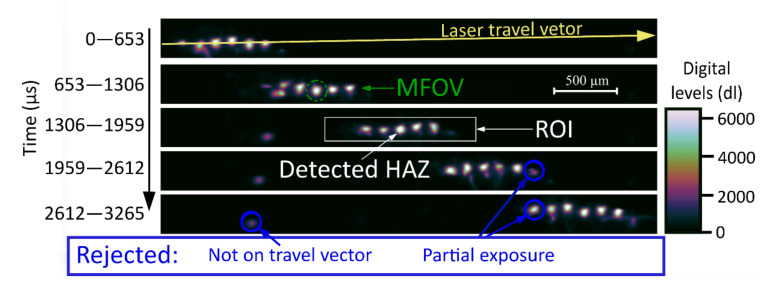
An example of a series of images captured using the setup in Figure 2. Each image is subsequent to the image above it in time, shown in µs on the left. An example Region of Interest (ROI) for a detected Heat Affected Zone (HAZ) is displayed in the white box. The MFOV is displayed as a green circle in the second image. Examples of three rejected hot spots are illustrated, highlighted with blue arrows, and circled.

The build laser is a pulsed system. Each exposure of the laser creates a distinct HAZ in the scene. Multiple HAZs are imaged within a single exposure of the camera, forming a straight line of hot spots on the laser travel vector. An automated HAZ detection algorithm was developed to identify the HAZs that met the detection criteria. The method involved a 29 × 195 pixel Region of Interest (ROI) that was cropped within the image and added to a historical database of ROIs for each video frame. Figure 4 shows the ROI detection process. The algorithm searched for regions of high signal level within an image and used the measured laser travel vector to reject ejected particles and other anomalous readings.

The HAZs at the beginning and end of the exposure were less intense than the central laser exposures. The physical explanation for this behavior is that only a portion of the detectable heating and cooling cycle was acquired. We can infer from this behavior that an entire cycle of heating and cooling occurred for the uniform central HAZs of each image. The detection algorithm required at least three detected HAZs for each ROI added to the database: the central HAZ of interest and one HAZ to each side. Any detected HAZ with a missing adjacent detected HAZ was rejected from the database. It can, therefore, be asserted that the entire thermal cycle was present for each central HAZ in the database. Dark images were subtracted from the acquired images prior to processing with the detection algorithm.

### 2.3. Thermal Field Reconstruction

**Figure 5:**
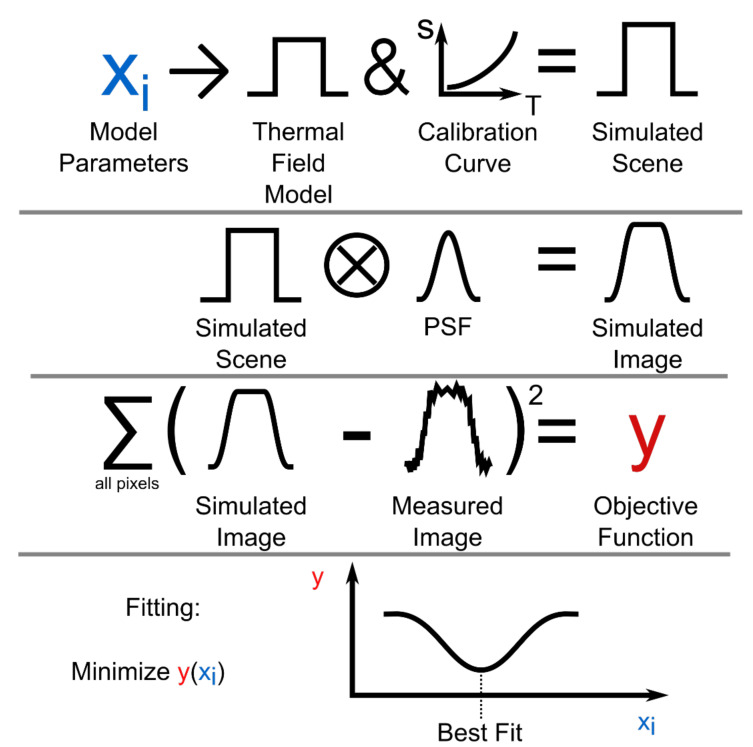
Workflow for application of thermal field reconstruction technique. The Point Spread Function (PSF) is a representation of the spatial transfer function of the thermographic instrument.

The standard or direct method of converting infrared images to thermal field measurements is to map the pixel values directly to temperature via a calibrated lookup table or function [6,7]. Figure 5 illustrates the steps required to implement a thermal field reconstruction technique that accounts for the spatial transfer function of the instrument. We utilized this technique to reconstruct the mean thermal field of the HAZ from the condensed measured image.

### 2.4. Instrument Characterization

The thermographic instrument was calibrated against a commercial blackbody source (AMETEK Land P1500) of known radiance. Blackbody calibration provides the mapping between measured signal and radiance. This mapping is only strictly valid for a uniform source that is of the same size and shape as the calibration source. The temperature of the blackbody source was measured using a calibrated IRT (AMETEK Land Cyclops L100) giving the blackbody measurement traceability to the ITS-90 temperature scale [24].

**Figure 6:**
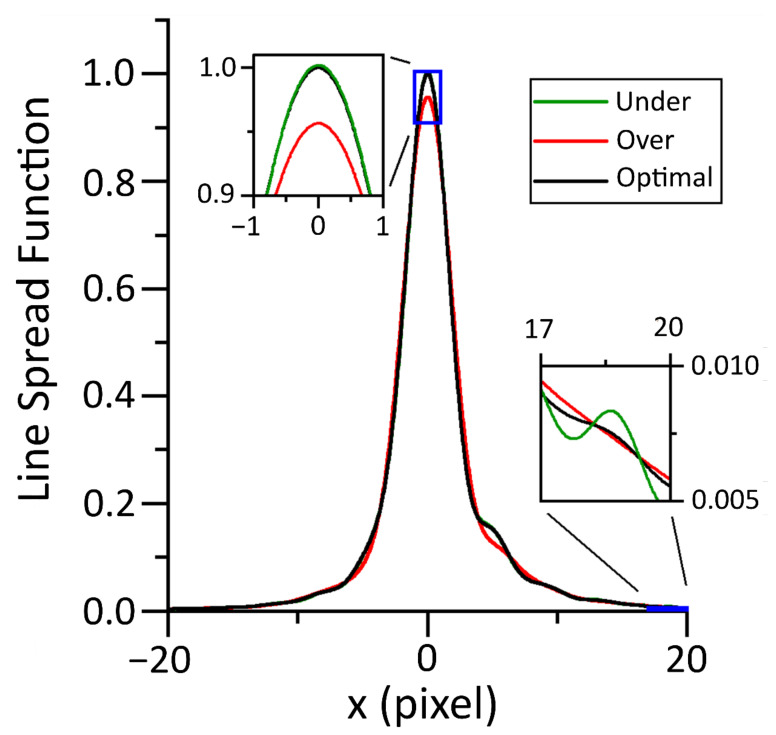
A frequency domain filter was used to smooth the knife edge data for differentiation. The parameters chosen for the filter effect the central peak and the low-amplitude wings of the distribution. The central peak and low-amplitude wings of the curve are shown zoomed in for clarity.

A slanted knife edge [17] was used to characterize the spatial transfer function of the thermographic instrument. See Section 3.2 for more details. The knife edge was placed between the thermographic instrument and the blackbody furnace at the same distance from the instrument as the HAZ during the L-PBF acquisition, and the data were smoothed with a frequency domain filter shown in Figure 6. A 3 mm diameter aperture was placed in the same plane as the knife edge to minimize scatter in the optical system. The exposure time of the instrument was adjusted to maximize the signal without approaching saturation. Then, 200 images were averaged to minimize noise, and dark images were removed. No correction for inhomogeneity in the sensitivity of the instrument was made.

The spatial transfer function was ascertained from the knife edge images by linearization [10], differentiation, and Abel inversion of the knife edge data. Numerical differentiation required the data to be smooth, smoothing the data required minimizing perturbations to the underlying form while removing as much noise as possible. A discrete Fourier-transform filter was used with zero attenuation from stationary to a spatial frequency of 0.1 per pixel followed by a one-tailed Gaussian roll off with a width of σ = 0.2 pixel per pixel. Figure 6 illustrates how the filtering parameters affect the shape of the measured derivative.

### 2.5. Validation

A series of circular ‘pinhole’ apertures (Thorlabs, PHW16) of uniform radiance were used to validate the performance of the reconstruction technique. The pinholes were placed in front of the blackbody cavity at the same distance from the instrument as the HAZ in the L-PBF acquisition. The temperature of the blackbody reached a steady state at 1188.8 °C. The temperature was monitored with a calibrated IRT, and the drift of the target was found to be less than 0.2 °C. The exposure time of the instrument was adjusted once to maximize the signal at the largest aperture without approaching saturation. A series of 200 images were averaged to produce the measured image. The validation targets were used to assess the performance of the reconstruction technique on known scenes approximating those observed in the L-PBF data. The accuracy of the recorded temperature at the centroid of the image was used to compare the performance of the reconstruction technique with the direct method.

## 3. Results

### 3.1. Blackbody Calibration

A standard approximation for the integrated spectral response of a radiometric temperature measurement system is the Sakuma–Hattori [25] equation:

(1) $$S_{m}=\varepsilon C{\left(\exp\left(\frac{c_{2}}{\mathrm{AT}+B}\right)-1\right)}^{-1}$$

where $S_{m}$ is the signal measured from a Lambertian surface [3] emitting at absolute thermodynamic temperature, T. A, B, and C are coefficients fitted to the signals measured from the blackbody furnace at a series of known T, their physical significance is explained in reference [26]. The emissivity (ε) is a property of the surface, which is assumed to scale the blackbody radiance. During calibration this is fixed at unity. $c_{2}$ is the second radiation constant [27]. A 0.5 mm diameter clear aperture was used to calibrate the device for its use in the L-PBF acquisition. During calibration, the T of the blackbody source and, therefore, the radiance of the scene remained constant throughout an exposure of the camera. For the L-PBF data, the temperature within one exposure varied significantly. Data acquired from the L-PBF process cannot be considered temporally resolved. We must, therefore, consider the signal levels in a measured image as the time-integrated radiance of the scene.

It is a limitation of the acquired data that the thermal fields cannot be considered stationary within a single exposure. This limitation does not affect our assertions about reconstruction of the spatial distributions of the thermal fields. A temporal temperature distribution must be assumed to demonstrate the effect of the spatial thermal field reconstruction technique for L-PBF in terms of temperature. We have assumed that the thermal field was stationary for 5 µs within a single exposure. The modeled signal was scaled by the ratio of assumed exposure time ($\Delta t_{m}$) to calibration exposure time ($\Delta t_{cal}$) to account for the decrease in signal due to the duration of the thermal field being less than the exposure time. The effective temperature (T*) as reported in the results section is as follows:

(2) $$T*=\frac{c_{2}}{Aln\left(\Delta t_{m}\varepsilon C/\Delta t_{cal}S_{m}+1\right)}-\frac{B}{A}$$

A more sophisticated temporal model of the thermal fields could be employed to better estimate the true peak temperatures and thermal gradients, but this is beyond the scope of this work. An assumed effective ε of 0.5 [9] was chosen to reconstruct the thermal fields from the L-PBF data. It is likely that uncertainty in this assumption will be a major factor in any final assessment of uncertainty in the inferred surface temperature. The true time- and temperature-dependent effective emissivity of the surface is beyond the scope of this work. This assumption has allowed us to demonstrate the need for a thermal field reconstruction method in L-PBF.

### 3.2. Spatial Transfer Function

The spatial transfer function of a thermographic instrument is commonly assumed to be independent of the composition of the scene and independent of the location within the total field of view [11,12,16]. Making these assumptions we can then assert that the measured image ($S_{m}$) of a ‘true’ radiance scene (S) can be modeled by

(3) $$S_{m}\left(x,y\right)=\iint S\left(\tilde{x},\tilde{y}\right)\mathrm{PSF}(x-\tilde{x},y-\tilde{y})d\tilde{x}d\tilde{y}$$

(4) $$S_{m}=S⊗\mathrm{PSF}$$

where x,y are the spatial coordinates in the image, $\tilde{x},\tilde{y}$ are the spatial coordinates in the scene, and PSF is the point spread function. The double spatial integral is equivalent to the two-dimensional convolution operator (⊗).

The PSF is a representation of the spatial transfer function, which determines the magnitude and character of blur in the imaging system. The slanted knife edge method [11,28,29] is an established characterization technique for imaging devices. Images of a slanted knife edge were used to ascertain the one-dimensional Edge Spread Function (ESF) as a function of perpendicular distance from the knife edge at a sample frequency of 100 points per pixel. The Line Spread Function (LSF) is the spatial derivative of the ESF:

(5) $$\mathrm{LSF}=\frac{d\mathrm{ESF}}{dx}$$

Moreover, the LSF is a one-dimensional projection of the PSF:

(6) $$\mathrm{LSF}(x)=\int \mathrm{PSF}\left(x,y\right)\;dy$$

**Figure 7:**
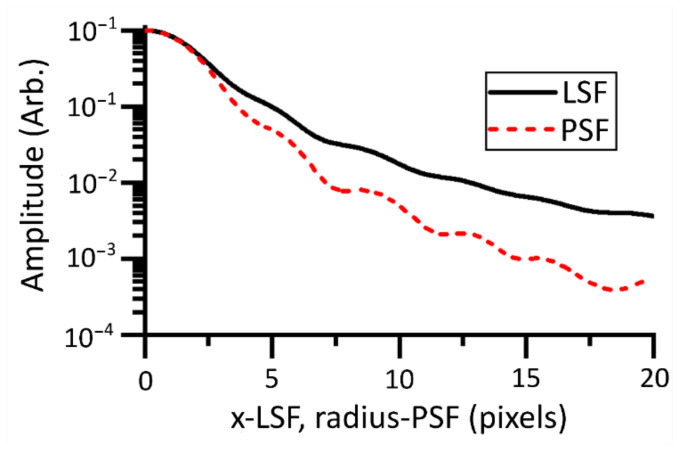
Numeric Abel inversion was used to convert the LSF (one dimensional in x) to the PSF (one dimensional in radius). The PSF has reduced by 3 orders of magnitude within 20 pixels.

The PSF can be ascertained from the LSF by applying the inverse Abel transform [30]. In a departure from the work by Lane et al. [10], we utilized numerical methods to ascertain the PSF from measurements of the LSF, instead of fitting a model directly to the ESF data and applying an algebraic inverse Abel transform. A numeric inverse Abel transform [30] implemented in MATLAB [18] was used to invert the spatial integral to ascertain the radial PSF. The performance of the numeric inversion was validated with modeled LSF data where the algebraic inverse Abel transform is calculable. The results of the numerical Abel inversion can be found in Figure 7.

There are various deconvolution methods [11,16,28] that attempt to reproduce the original scene without a priori assumptions of the form of the thermal fields required. A systematic assessment of the performance of these deconvolution techniques for reconstruction of scenes such as those found in L-PBF does not appear to be present in the literature. The reconstruction of the original scene by regularized deconvolution [10] did not accurately reproduce the validation scenes generated in this work.

Measurement FOV is an important concept as it defines how large a uniform-temperature object must be to allow the central pixel to make an accurate measure of its radiance. We have chosen to define the Measurement FOV of our instrument in a similar way to that of an IRT [18,19]. The Measurement FOV of an IRT is usually measured by a series of circular uniform apertures with varying diameters. The aperture technique relies on a plateau of signal vs. aperture dimeter. The Measurement FOV is the diameter at which the signal level is 95% or 99% of this plateau value. The poor, long-range, Size of Source Effect (SSE) performance of thermographic instruments [31,32] makes the determination of this plateau subjective. We instead used our measurement of the PSF to generate a simulated curve of the signal in the central pixel against a uniform circular scene diameter. This allows us to approximate a 100% signal level in the central pixel, from which we can ascertain the diameter that gives 95% of this signal level. This is our measure of the Measurement FOV. We calibrated the instrument with an aperture that was larger than the Measurement FOV while also being small enough not to incur any significant SSE scatter problems.

**Figure 8:**
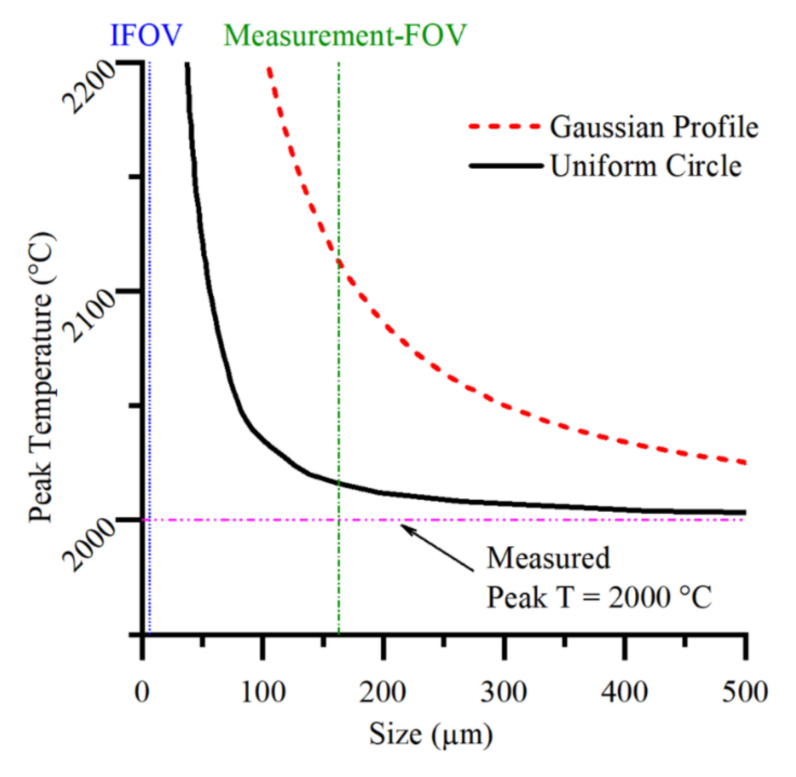
The size and temperature an object would be for our thermographic instrument to measure a peak temperature of 2000 °C. The size of the Gaussian profile is the full width at half maximum.

The peak temperature (measured by the direct method) could be generated by any distribution from within a broad class of thermal distributions. Figure 8 illustrates the relation between size and temperature that would give identical measured peak temperatures for two forms of thermal distribution. For nonuniform (Gaussian) temperature distributions, the size effects the measured temperature even when the size of the distribution is significantly larger than the Measurement FOV. The data in Figure 8 were generated by convolution of a series of rotationally symmetric scenes with the PSF of the instrument. The search algorithm ‘fminbnd’ in MATLAB was used to find the peak temperature that gave a measured peak temperature of 2000 °C at the central pixel, for each scene size.

### 3.3. Spatial Transfer Function

The inbuilt ‘fminsearch’ nonlinear iterative optimization algorithm in MATLAB, was used to find the thermal field model parameters that produced a simulated image most similar to the measured image. The thermal field model and, hence, the simulated scene were generated at subpixel resolution. Nine subpixel evaluation points were used to generate the thermal field models and the PSF. A two-dimensional convolution kernel describing the PSF was generated by rotational interpolation of the radial profile as a 157 pixel (1413 evaluation points) square. After the convolution process, the convolved image was pixelated by averaging over each pixel to create the simulated image. The two-dimensional convolution process is computationally expensive and limits the number of subpixel evaluation points that can be used to generate the simulated image.

The peak effective temperature and width of the distribution, which is assumed to be Gaussian, are free parameters. The adjacent HAZs are included in the model as they affect the signal levels of the central HAZ. The model parameters of the adjacent HAZs were allowed to vary independently of the central HAZ.

The thermal field of the validation targets was assumed to have a uniform circular shape. Two parameters were used in the thermal field model: the temperature and the radius. The position of the aperture within the image shifted from aperture to aperture by a small amount. The centroid of the aperture in the measured image was used to determine the center of the circular thermal field within the simulated scene.

The penultimate step in the workflow diagram in Figure 5 was the sum of the squared differences between each pixel of the simulated image and the measured image. This was defined as the objective function. Minimizing this value by varying the model parameters provided the most accurate model of the measured scene. The measured scene was used as a starting estimate for the minimization algorithm. A local minimum value was achieved, which was used as an approximation of the global minimum achieved with the model parameters.

## 4. Discussion

### 4.1. Infrared Images of Metal Powder Fusion by Pulsed Laser

The individual hot spots in Figure 4 clearly illustrate the pulsed nature of the build laser. Several distinct HAZs are present in each image, meaning the exposure time of the camera was too long to temporally discriminate the thermal fields. The minimum exposure time of the camera was determined by the FOV. A trade off was made between capturing sufficient passes of the laser and minimizing the exposure time. As the exposure time is reduced, the temporal resolution increases but the signal falls linearly, decreasing the overall signal-to-noise ratio and thermal resolution of the measurement.

**Figure 9:**
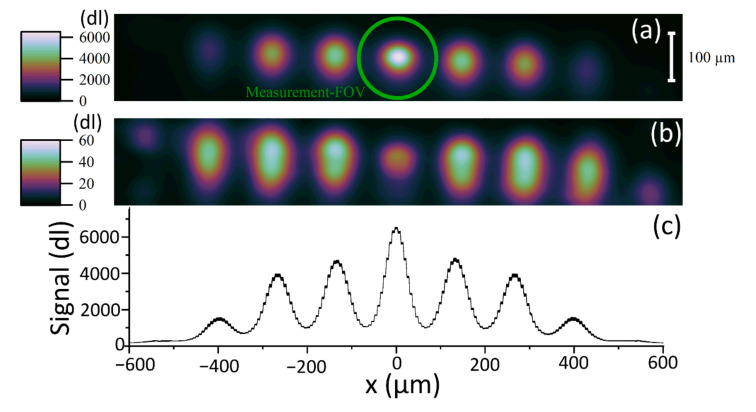
(**a**) Mean of 2156 183 × 29 pixel Regions of Interest (ROIs) around the central HAZ cropped from the captured images as per Figure 4. (**b**) Standard error in the mean of the 2156 ROI images making up the mean. (**c**) Line profile of (**a**) showing the 95% confidence interval as the vertical line thickness. The size of the Measurement FOV is included to illustrate that a correction for the spatial transfer function of the thermographic instrument must be made. The scale for (**a**,**b**) is shown in the top right and is in dl.

The mean of the 2156 ROIs that met the criteria of the HAZ detection algorithm is shown in Figure 9. The Measurement FOV is included in the figures to support our assertion that the thermographic instrument is unable to make an accurate, direct measure of the thermal fields from the infrared measured images. This fact is not self-evident from the acquired images, which appear to be well resolved. The high sampling rate (small pixels) of the instrument allows the use of a thermal field reconstruction technique.

### 4.2. Characterization Results

**Figure 10:**
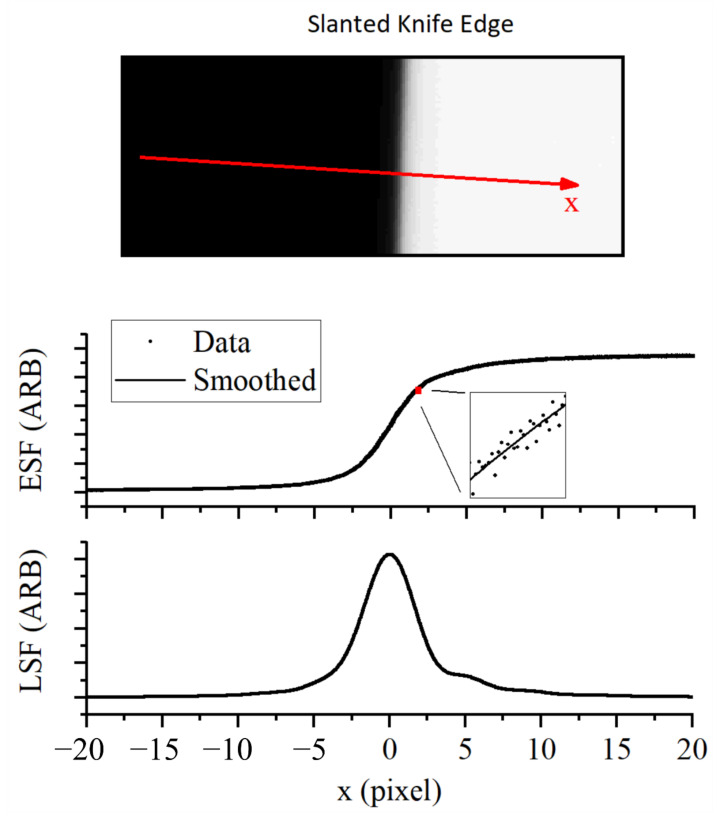
Image of a slanted knife edge used to measure the Edge Spread Function (ESF), the ESF was smoothed and spatially differentiated to provide a measure of the Line Spread Function (LSF).

The characterization data presented in Figure 10 show that the width of the LSF is several pixels, indicating that the system is oversampled. Oversampling in this context means that the spatial Nyquist frequency of the sensor is higher than the frequency of the cutoff MTF of the system. A cutoff MTF of 0.99 is used as suggested by a related metric in the literature [33]. The cutoff frequency for our instrument was found to be 0.009 pixels. The MTF was calculated by Fourier transform of the measured PSF. The Measurement FOV was measured by the method described in Section 3.2 to be a circle of 163 µm diameter.

The Measurement FOV measured is significantly larger than the expected size of the high-temperature part of the HAZ. For any instrument where the Measurement FOV is large or of a comparable size to the features of interest, the direct method of pixel to thermal field conversion should not be used. The slanted edge method provides a simple and accurate way to measure the spatial transfer function.

### 4.3. Validation Targets

**Figure 11:**
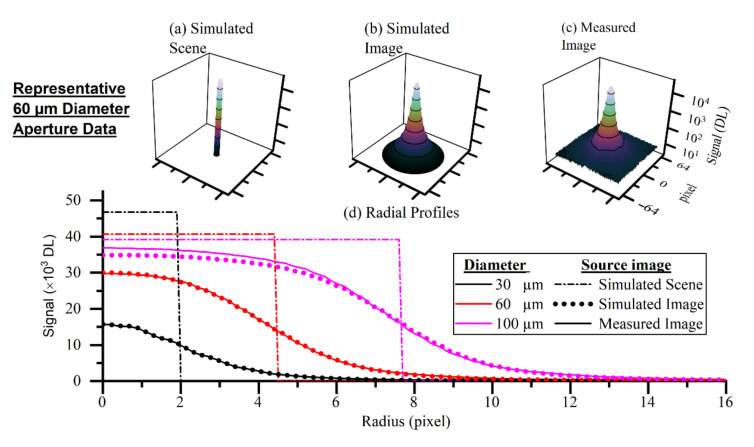
Representative data of the validation targets. (**a**) Fitted parameterized thermal field model; (**b**) convolution of (**a**) with the spatial transfer function of the thermographic instrument; (**c**) measured image of the 60 µm aperture; (**d**) radial profiles of three of the pinhole measured images along with the radial profiles of their corresponding simulated scenes and simulated images.

Figure 11 presents representative data considering the performance of the reconstruction technique when applied to the validation targets. The plots in Figure 11a,b show the simulated scene and simulated image generated from the best fit model parameters. The close alignment in the radial profiles of the simulated and measured images in Figure 11d demonstrates the validity of the characterization methodology. The discrepancy for the larger-diameter apertures can be partly explained by the inherent weighting of the perimeter of the scene. The amount of data on the perimeter scales with the radius of the target meaning the central plateau is weighted relatively less for the larger-diameter targets. A perfectly functioning reconstruction technique would generate thermal fields, and hence simulated scenes, with identical temperatures. Figure 11d illustrates the two extremes of aperture sizes where the technique is working as we anticipated.

**Figure 12:**
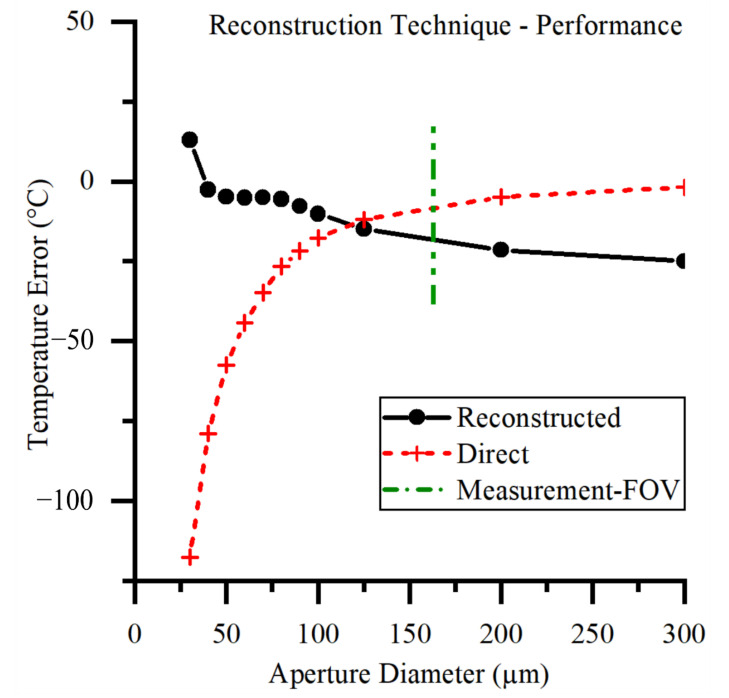
Accuracy of thermal field reconstruction for a series of uniform circular apertures. An idealized measurement or a perfect reconstruction technique would be a horizontal line at 0 °C error.

The improvements in accuracy of the measured thermal fields shown in Figure 12 are due to the application of the reconstruction technique. The temperature error, as measured at the center of the scene, is the metric chosen to compare the performance of the reconstruction technique with the direct method. This is comparable with the error in a single-point IRT detector. This is a stringent performance metric because the central pixel of the direct method reports the most accurate temperature. The chosen metric does not provide information about the improvement in accuracy for the rest of the distribution.

Validation of the reconstruction technique is required to give confidence that the reconstructed thermal field is closer to the true thermal field than that measured by the direct method. We can see from the results that the reconstruction technique performs well in the 30–100 µm aperture size. Above this size, the direct method performs sufficiently well without the need for a reconstruction technique to be applied.

### 4.4. Heat Affected Zone in L-PBF

**Figure 13:**
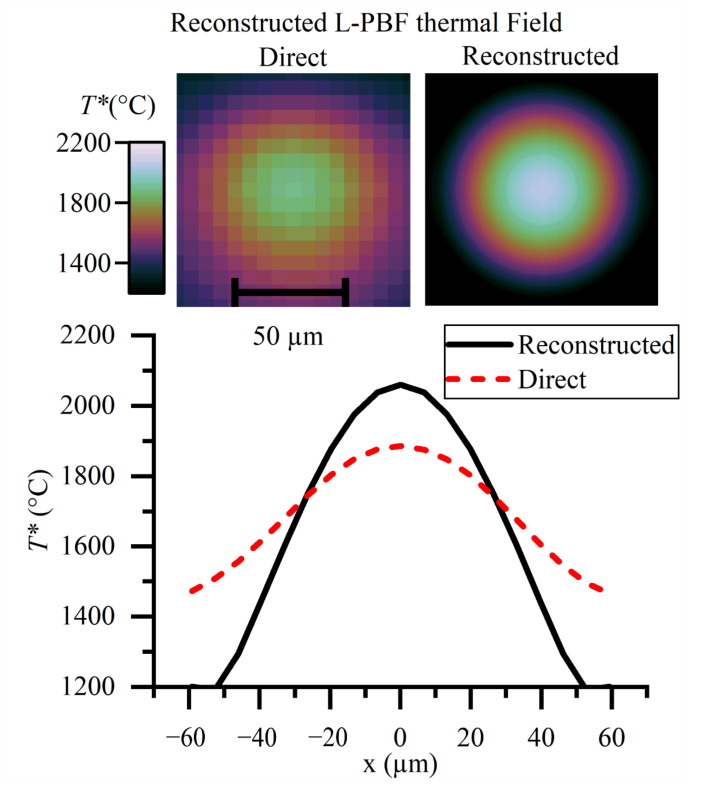
Reconstructed thermal field of the heat affected zone in laser-based powder bed fusion. The direct measure is made by conversion of the discrete pixel values to temperature. The reconstructed thermal field takes into account the spatial transfer function of the instrument. This work has shown the direct method to have a significant error for the length scales involved.

The measured thermal fields in Figure 13 illustrate the significant difference between the direct method and the reconstruction technique that has been articulated throughout this paper. The technique makes a 174 °C difference to the peak measured T*. The spatial thermal gradients are significantly higher than would be inferred from the direct method. This is important because the solidification rate is proportionate to the thermal gradient. It is a limitation of this work that the infrared images cannot be considered temporally resolved, making truly quantitative measurements of temperature beyond the scope of this work. The same methodology applies to any other thermographic applications where the feature sizes are small compared with the optical resolution of the instrument.

## 5. Conclusions

In this work, we have shown that it is possible to overcome the low measurement resolution of a typical thermographic instrument and produce a more accurate temperature map than the camera can natively produce due to the inherent defects introduced by optics quantitatively described by the PSF. We have demonstrated that it is possible to obtain accurate thermal field measurements in the heat affected zone during L-PBF through the determination of the spatial transfer function. A technique to reconstruct the thermal field from oversampled, but unresolved, infrared images has been provided and validated. The methodology reproduced validation data with significantly improved accuracy over the standard (direct) method of thermal field measurement. This approach can be applied to other thermal metrology applications with a disparity between MFOV and the size of the HAZ, which is common in additive manufacturing applications.

A standardized approach to the definition of the measurement field of view for a thermographic instrument would be a significant advantage in the field of quantitative thermography for additive manufacturing. A standardized approach will give researchers wishing to measure thermal fields an indication as to whether the direct method can be used. The decision can be made on whether a reconstruction technique must be applied. Future analysis of the data collected during this experiment may yield the temporally resolved temperature of the HAZ, but this was beyond the scope of this work because the methodology specifically focused on affects effecting spatial resolution.

We have suggested a method for determining the measurement field of view which may be utilized for these purposes. Quantitative measurements of the thermal fields in the heat affected zone for additive manufacturing remains a challenging task. The tools provided in this work improves the ability to make truly quantitative measure of temperature in challenging environments.

## Figures and Tables

**Table 1:** The build parameters and imaging parameters used during the image acquisition.

Build Parameters		Imaging Parameters	
Laser Exposure time	56 µs	Exposure time	653 µs
Mark Spacing	135 µm	Frame rate	1600 fps
Laser Peak Power	150 W	IFOV	6.58 µm
Hatch Spacing	40 µm	Wavelength band	0.85–1.00 µm
Material	CM247	#pixels	128 × 2048

## Data Availability

All relevant data is shown in the paper or could be re-created by following the methodology in the paper.
